# Effects of Mild Cognitive Impairment on the Event-Related Brain Potential Components Elicited in Executive Control Tasks

**DOI:** 10.3389/fpsyg.2018.00842

**Published:** 2018-05-29

**Authors:** Montserrat Zurrón, Mónica Lindín, Jesús Cespón, Susana Cid-Fernández, Santiago Galdo-Álvarez, Marta Ramos-Goicoa, Fernando Díaz

**Affiliations:** ^1^Laboratorio de Neurociencia Cognitiva Aplicada, Departamento de Psicoloxía Clínica e Psicobioloxía, Facultade de Psicoloxía, Universidade de Santiago de Compostela, Santiago de Compostela, Spain; ^2^Basque Center on Cognition, Brain and Language, San Sebastián, Spain; ^3^Sezione di Neuroscienze Cognitive – Laboratorio di Neurofisiologia, IRCCS Centro San Giovanni di Dio, Fatebenefratelli, Brescia, Italy

**Keywords:** mild cognitive impairment, event-related brain potentials, Stroop task, Simon task, Go/NoGo task

## Abstract

We summarize here the findings of several studies in which we analyzed the event-related brain potentials (ERPs) elicited in participants with mild cognitive impairment (MCI) and in healthy controls during performance of executive tasks. The objective of these studies was to investigate the neural functioning associated with executive processes in MCI. With this aim, we recorded the brain electrical activity generated in response to stimuli in three executive control tasks (Stroop, Simon, and Go/NoGo) adapted for use with the ERP technique. We found that the latencies of the ERP components associated with the evaluation and categorization of the stimuli were longer in participants with amnestic MCI than in the paired controls, particularly those with multiple-domain amnestic MCI, and that the allocation of neural resources for attending to the stimuli was weaker in participants with amnestic MCI. The MCI participants also showed deficient functioning of the response selection and preparation processes demanded by each task.

## Introduction

Here we report the findings of several studies carried out by our research group in which we recorded event-related brain potentials (ERPs) elicited in participants with mild cognitive impairment (MCI) during performance of executive tasks. The aim of this research was to search for neurofunctional indexes of executive processes which can be used in the diagnosis of MCI. This might contribute to the early detection of Alzheimer’s disease (AD), of enormous socio-sanitary importance, and to halting progression of the disease by the timely application of appropriate treatments.

In section “Executive Functions, Tasks, Behavior and Brain Electrical Activity,” we describe the three executive tasks, i.e., the Stroop, Simon, and Go/No-Go tasks, which we have adapted to induce ERPs. We also describe the characteristic ERP components elicited in these tasks and the cognitive processes associated with each. In section “Aging, Mild Cognitive Impairment (MCI), Performance of Executive Tasks and ERPs,” we define the different subtypes of MCI and summarize the results obtained in our studies on the effect of MCI on the ERP components in the three tasks.

## Executive Functions, Tasks, Behavior and Brain Electrical Activity

The term executive function encompasses those brain functions that enable us to set goals and control the skills and behaviors required to achieve these goals. These functions include a series of cognitive processes, including attention, cognitive control, working memory, cognitive inhibition, and flexibility, which we use in our activities of daily living to monitor behaviors and implement goal-directed actions (Chan et al., 2008; Diamond, 2013).

The study of brain functions that support executive processes has traditionally been carried out by analyzing the behavioral responses to classic executive tasks in patients with some type of brain lesion or alteration. More recently, functional neuroimaging techniques have been used to record the haemodynamic activity in such patients and healthy controls while they perform executive tasks (see Gilbert and Burgess, 2008; Verdejo-García and Bechara, 2010; Lezak et al., 2012). These studies have highlighted the role of prefrontal areas in executive functioning, but do not provide any information about the temporal course of brain activity associated with executive processes. The ERP technique is a valuable tool in this respect, as it enables direct measurement of brain functioning, with a high temporal resolution (milliseconds), is non-invasive (ERPs are recorded with electrodes attached to the intact scalp), simple to use and relatively inexpensive. ERPs are positive and negative deflections of voltage that are considered components of brain activity and provide information about the temporal dynamics of the different stages of stimulus processing (perception, evaluation, categorization) and response processing (selection, preparation, and control) (Luck, 2005).

Event-related brain potential components can be characterized according to their amplitude (in microvolts, μV), latency (in milliseconds, ms) and topographical distribution. Various executive tasks have been adapted for use during the recording of brain electrical activity, in order to identify the ERP components associated with executive functioning. We have adapted three such tasks for this purpose: the Stroop, Simon, and Go/NoGo tasks.

All three tasks allow the study of attentional control, as subjects must direct their attention toward the characteristics of the stimulus that are relevant to the task demands and inhibit responses that are incompatible with the demands. Thus, the N2 and P300 components have been identified in relation to stimulus processing. These components are associated with the processes of directing and controlling attention to relevant stimuli: N2/N2b is associated with evaluation (Ritter et al., 1979; Folstein and Van Petten, 2008) and P300/P3b with categorization (Donchin, 1981; Polich, 2007) of the stimuli, although these components have specific characteristics in each task. Regarding the response processing, the lateralized readiness potential (LRP) is obtained in all three tasks and is generated within the motor cortex. The LRP provides information about the timing of response selection (stimulus-locked LRP; sLRP) and of planning and executing the response (response-locked LRP; rLRP) (Smulders and Miller, 2012).

### Stroop Task

**FIGURE 1:**
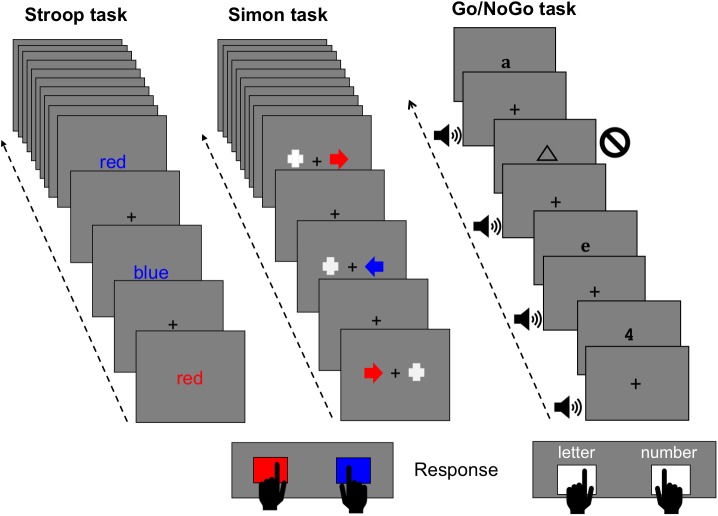
Outline of the Stroop, Simon, and Go/NoGo tasks used in our studies.

All of the different versions of the Stroop task available compare the performance (reaction times -RT- and hits) under two conditions (**Figure 1**): one in which there is conflict between two types of information, one relevant and the other irrelevant to the task (e.g., the word “red” displayed in blue -incongruent stimulus-), and another that does not produce conflict (e.g., the word “red” displayed in red -congruent stimulus- or colored X-strings). Participants are usually asked to respond to the color of the stimulus and to ignore the word, thus generating the so-called Stroop effect, whereby the RT is longer in the conflict condition than in no-conflict condition. Considering that words induce an automatic reading response, the Stroop effect is attributed to interference generated by the meaning of the incongruent word in the task of responding to the color of the stimulus (see Stroop, 1935; MacLeod, 1991).

**FIGURE 2:**
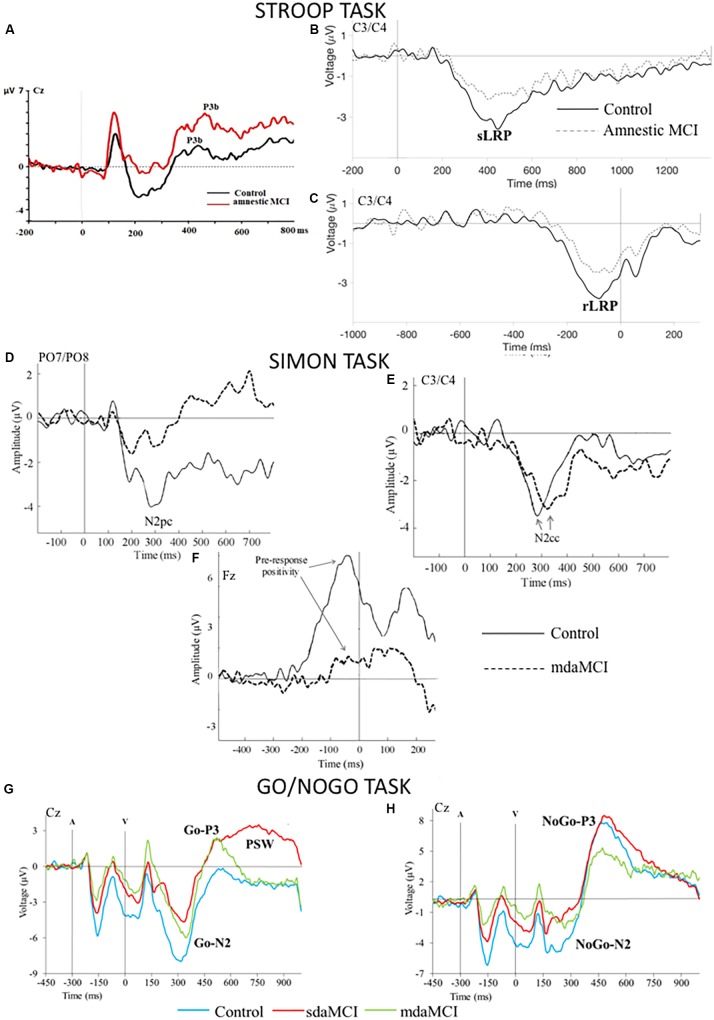
Grand average Event-Related Brain Potentials (ERPs) waveforms elicited by **(A)** the incongruent stimuli in the Stroop task, **(B,C)** the response processing in the Stroop task [**(B)** the 0 ms point corresponds to the presentation of the stimulus, **(C)** the 0 ms point corresponds to the emission of the response], **(D,E)** the stimuli in the Simon task, **(F)** the response processing in the Simon task; the 0 ms point corresponds to the emission of the response, **(G,H)** the Go and NoGo visual stimuli (V) in the Go/NoGo task (A: tone prior to visual stimulus).

The ERP waveforms recorded in young and old healthy participants while they are processing color-word stimuli basically include (**Figure 2**) the frontal-central N2 and the parietal P3b (Zurrón et al., 2009, 2013, 2014).

The frontal-central N2 component is characteristic of tasks in which a conflict must be resolved before the response is given (N2 is larger for higher conflict) and it has therefore been associated with cognitive control processes (see review by Folstein and Van Petten, 2008).

The parietal P3b component (or P300) is observed in attentional tasks when an informative task-relevant stimulus is detected, and the P3b amplitude is thus larger for target than non-target stimuli (Donchin, 1981; Polich, 2007).

The cognitive processes associated with N2 and P3b involve respectively evaluation and categorization of the stimulus. Indeed, N2 and P3b latencies are considered indicators of the time required to evaluate and categorize the stimulus for resolution of a task (Kutas et al., 1977; Hillyard and Kutas, 1983; Folstein and Van Petten, 2008). The P3b amplitude is smaller for incongruent stimuli than for congruent stimuli in young participants, and this difference has been considered an indicator of the semantic conflict that occurs in the former relative to the absence of such conflict in the latter (Zurrón et al., 2009).

### Simon Task

In the Simon task (**Figure 1**), participants are required to respond to a non-spatial feature (i.e., color, shape) of a lateralized stimulus by pressing one of two response buttons that are lateralized in the same spatial arrangement. Although the stimulus position is irrelevant to the task, the RT is longer when the response side is spatially incompatible with the stimulus position than in trials that require an ipsilateral response regarding the stimulus position. This spatial interference, known as the Simon effect, is evoked by visual (Craft and Simon, 1970), auditory (Simon and Small, 1969), and somatosensory (Hasbroucq and Guiard, 1992) stimulation, regardless of whether the participants respond with hand, feet or eye movements (Leuthold and Schröter, 2006).

This paradigm enables us to study the spatial attention given to the target and to inhibition of the non-target stimulus, the cognitive control used to suppress the spatial tendency of the response and the motor processes involved in the response preparation. ERPs can be used to study these cognitive processes (**Figure 2**). The contralateral posterior negativity (N2pc), a correlate of visuospatial attention to the lateralized target stimulus and suppression of the non-target stimulus (Luck and Hillyard, 1994; Eimer, 1996), arises from extrastriate visual areas (Luck et al., 1997; Hopf et al., 2000). The contralateral central negativity (N2cc) is generated in the dorsal premotor cortex during activity involved in preventing spatial responses (Praamstra and Oostenveld, 2003; Praamstra, 2006; Cespón et al., 2012). N2pc and N2cc appear between 200 and 300 ms post-stimulus in young adults. The rLRP component is associated with response preparation.

### Go/NoGo Task

In the Go/NoGo task (**Figure 1**), participants are usually required to respond by pressing a button in response to frequent stimuli (Go stimuli), while withholding the response to different infrequent stimuli (NoGo stimuli). This task is considered an executive function task (Rubia et al., 2001), and it can be used to study several cognitive processes such as stimulus evaluation, response inhibition and response control (Botvinick et al., 2001; Lucci et al., 2013).

The ERP correlates of the Go stimuli evaluation processes are N2b and P3b, although they are often called Go-N2 and Go-P3 when obtained with this task (**Figure 2**). In young adults, N2b (latency: 200–300 ms post-stimulus) shows maximum amplitude at central electrodes and P3b (300–500 ms post-stimulus) at parietal electrodes. In addition, the sLRP and rLRP components have been studied in relation to response processing in the Go condition.

With NoGo stimuli (**Figure 2**), the NoGo-N2 and NoGo-P3 ERP components are identified at frontocentral locations around 200–400 and 300–500 ms post-stimulus, respectively (Pfefferbaum and Ford, 1988; Jodo and Kayama, 1992; Falkenstein et al., 2002; Vallesi et al., 2009). Both components have traditionally been considered correlates of response inhibition (e.g., Jackson et al., 1999; Bokura et al., 2001; Nakata et al., 2009).

## Aging, Mild Cognitive Impairment (MCI), Performance of Executive Tasks and ERPs

Executive functioning is known to decline in healthy cognitive aging (Park and Schwarz, 2002; Raz et al., 2005; Reuter-Lorenz and Park, 2010; Grady, 2012; Harada et al., 2013; Kirova et al., 2015). In Stroop tasks, the RT is slower and the Stroop effect is larger in older than in young people (see MacLeod, 1991; Van der Elst et al., 2006; Peña-Casanova et al., 2009); the Simon effect increases in normal aging (Proctor et al., 2005; Juncos-Rabadán et al., 2008), and an age-related decline in behavioral responses has been observed in Go/NoGo tasks (Falkenstein et al., 2002; Lucci et al., 2013; Correa-Jaraba et al., 2016; Hsieh et al., 2016).

The ERPs elicited in the three tasks are sensitive to cognitive decline in healthy aging, and, relative to younger participants, healthy older participants display longer N2 and P3b latencies and smaller parietal P3b amplitude in Stroop (Zurrón et al., 2014) and Go/NoGo tasks (Falkenstein et al., 2002; Vallesi, 2011), as found in oddball tasks (Polich, 1997); longer N2pc in the Simon task (Van der Lubbe and Verleger, 2002; Cespón et al., 2013a), as also observed in visual search or selection tasks (Lorenzo-López et al., 2008, 2011; Amenedo et al., 2012; Wiegand et al., 2013, 2015); and delayed rLRP onset and larger sLRP amplitude in the Simon task (Cespón et al., 2013a), in consonance with previous findings (Yordanova et al., 2004; Kolev et al., 2006; Roggeveen et al., 2007; Wild-Wall et al., 2008; Wiegand et al., 2013). In addition, the P3b distribution is frontal in older and parietal in younger participants in the Stroop task (Zurrón et al., 2014), and the central NoGo-P3 amplitude is larger in older than in younger participants (Vallesi, 2011).

Some middle-aged and old adults show greater cognitive decline than expected according to their age and educational level, although their independence in daily life activities is preserved and they do not meet the criteria for diagnosis of dementia. This intermediate stage between normal cognitive aging and dementia is denominated MCI (Petersen et al., 1999; Knopman and Petersen, 2014). Interest in MCI has increased in recent years because the condition is associated with a higher risk of progressing to dementia (Winblad et al., 2004). MCI is frequently (although not always) associated with a decline in memory functioning, which may also be accompanied by deterioration in other cognitive functions, including executive functions. Four subtypes of MCI are currently distinguished (Petersen, 2016): single-domain amnestic MCI (sdaMCI, characterized by memory impairment only), multiple-domain amnestic MCI (mdaMCI, characterized by impairment in memory and in other cognitive domains), single-domain non-amnestic MCI (sdnaMCI, preserved memory but an overt decline in another cognitive domain), and multiple-domain non-amnestic MCI (mdnaMCI, preserved memory but with evidence of decline in several cognitive domains). Individuals with amnestic MCI (aMCI), especially those with mdaMCI, display a greater risk of developing AD than the healthy old population (Petersen et al., 1999, 2009).

In our ERP studies involving administration of Stroop, Simon and Go/No-Go tasks to participants older than 50 years (aMCI participants and healthy controls matched for age and years of schooling), we found that the effects of aMCI on the behavioral responses depended on the task used (Cespón et al., 2013b, 2015a; Cid-Fernández et al., 2014, 2017a,b; Ramos-Goicoa et al., 2016). However, aMCI affected the latencies and amplitudes of ERP components associated with executive functioning in all three tasks (**Figure 2**).

In the Stroop task, aMCI did not affect behavioral responses, probably because the task was simpler (only two types of response) than the standard versions of the Stroop test. In contrast, the latency of P3b component generated by congruent and incongruent stimuli was longer in middle-aged aMCI participants than in the paired controls; however, no differences between groups were observed in response to colored X-strings, which were presented separately and did not mobilize executive processes. aMCI affected the P3b amplitude: we observed a greater difference in P3b amplitudes between conditions (colored X-strings versus congruent or incongruent stimuli) in aMCI participants than in healthy controls. As only the color of the X-string stimuli, but both the color and semantic meaning of the congruent and incongruent stimuli must be evaluated, this finding may be attributed to a greater effect of reading-related interference in aMCI participants than in the controls. Finally, the sLRP and rLRP amplitudes were smaller in aMCI participants than in healthy controls (Ramos-Goicoa et al., 2016).

In the Simon task, the error rate (but not the RT) was affected by mdaMCI, indicating greater interference due to the spatial position of the stimulus in participants with mdaMCI than in healthy controls. In relation to stimulus processing, we observed that the latencies of the N2 and N2cc components were longer in the mdaMCI participants than in the paired controls and attributed these findings to respectively a longer time devoted to stimulus evaluation processes and delayed allocation of neural activity associated with cognitive control of the spatial response (Cespón et al., 2013b, 2015a,b). The N2pc amplitude was smaller in mdaMCI participants than in the controls, and as N2pc is an ERP correlate of the direction of the spatial attention to lateralized stimuli (Luck and Hillyard, 1994; Woodman and Luck, 1999; Hickey et al., 2009), we conclude that the mdaMCI participants show a deficit in neural resources allocated to spatial attention.

In ERPs related to response processing in the Simon task (Cespón et al., 2013b, 2015b), the rLRP amplitude was smaller in sdaMCI and mdaMCI participants than in controls, and the amplitude of the frontocentral pre-response positivity component between 80 and 20 ms pre-response (Nessler et al., 2007) was smaller in mdaMCI and sdaMCI participants than in controls.

In the Go/NoGo task, MCI participants (mainly mdaMCI) showed slower responses and fewer hits in response to Go stimuli than the controls.

The latency of the N2-Go component was longer in mdaMCI participants than in controls (Cid-Fernández et al., 2017a). This finding is consistent with those reported by Mudar et al. (2016) for a semantic-type Go/NoGo task, in which the latencies of the Go-N2 and NoGo-N2 components were longer in aMCI participants than in controls. We also observed smaller Go-N2 amplitude in sdaMCI participants than in the paired controls, as well as a smaller NoGo-N2 amplitude in sdaMCI and mdaMCI participants than in the controls. López Zunini et al. (2016) observed smaller Go-P3 and NoGo-P3 amplitudes in aMCI participants than in controls. Therefore, aMCI participants display a deficit in neural resources dedicated to the Go and NoGo stimuli. These deficit in neural resources may be unspecific, as they affect both stimuli, or they may be related respectively to evaluating and categorizing the target stimulus and to inhibiting predominant responses not relevant to the task demands.

A late positive slow wave (PSW) was identified in the 700–1000 ms interval after the Go stimulus in sdaMCI participants, and it was absent in healthy controls and subjects with mdaMCI (Cid-Fernández et al., 2017a). This ERP component overlaps with the Go-P3 and is thought to indicate additional operations after categorization of target stimuli. In the sdaMCI participants, this component may indicate compensatory operations in order to maintain acceptable levels of performance, as the behavioral response of this subgroup of participants was similar to that of the controls. Furthermore, this hypothesis was consistent with the longer sLRP latency in response to Go stimuli in sdaMCI participants than in the controls (Cid-Fernández et al., 2017b). In this study, we also observed that the amplitude of the sLRP component was smaller in mdaMCI participants than in healthy controls.

In summary, our findings on the components of the ERPs elicited in executive tasks indicate that, relative to age-matched controls, aMCI participants exhibit (a) longer latencies, indicating slower neural functioning in the stimulus and response processing, (b) changes in the amplitudes of the N2 and P3b components, reflecting fewer neural resources allocated for attending to the relevant dimension of the stimulus, and (c) smaller LRP amplitudes, indicating a lower capacity of the motor cortex to allocate neural resources for selection and preparation of the motor response. The decline in processing associated with aMCI is greater than in healthy aging and is more evident in mdaMCI than in sdaMCI participants.

In light of the above, we conclude that aMCI participants present neurofunctional alterations in executive functioning, independently of any behavioral responses, and we believe that the neurofunctional deficits appear prior to behavioral deficits in aMCI participants. Exploration of the sensitivity and specificity of the ERPs components is therefore of interest regarding the potential use of these components as markers of MCI and possible predictors of AD.

## Author Contributions

MZ, ML, JC, SC-F, SG-Á, and FD: wrote the manuscript. JC, MR-G, and SC-F: recorded the brain electrical activity. MZ, ML, JC, SC, SG-Á, MR-G, and FD: analyzed the ERP waveforms and identified the different components. MZ, JC, SC-F, SG-Á, and MR-G: carried out statistical analysis of the data.

## Conflict of Interest Statement

The authors declare that the research was conducted in the absence of any commercial or financial relationships that could be construed as a potential conflict of interest.
